# Supplementary material for: Agent-based modeling of macrophage-fibroblast interactions in the immune response to biomaterials
Source: PLoS One. 2025 Aug 19;20(8):e0329186. doi: 10.1371/journal.pone.0329186 (PMC12364366; doi:10.1371/journal.pone.0329186)
Supplement: S1 File — (PDF) [file pone.0329186.s001.pdf]

Agent-Based Modeling of Macrophage-Fibroblast Interactions in the Immune Response to Biomaterials

Supplementary Materials

Jennifer Riccio, Luca Presotto, Shir Bahiri, Liad Doniza, Donato Inverso, Laura Sironi, Uri Nevo, Giuseppe Chirico

Fig S1 shows, from left to right and from top to bottom, M1, M2 and F2 activation variables, the total number of cells, the amount of pro- and anti-inflammatory mediators and the number of newly recruited cells, in two different scenarios, a washout (a) and a continuous exposure of PIM (b). Notably, we found that the kinetics profiles were similar between continuous PIM stimulation and the washout condition, suggesting that the system's response was largely unchanged by the removal of PIM at the chosen time point. This suggests that a 12-hour exposure to PIM is sufficient to initiate a self-sustaining inflammatory cascade, driven by local interactions and internal feedback mechanisms. Such behaviour highlights the robustness of the model in capturing essential features of the immune response, as the dynamics remain stable even without ongoing external stimulation. However, it is likely that a significantly shorter exposure to PIM would more substantially alter cellular dynamics.

Bar plots in Fig S2 represent the outcomes of the sensitivity analysis performed for the ODE model by Maiti, with each bar indicating the magnitude of influence that individual parameters have on the model's behaviour. More in detail, we evaluated the effect that the variation of each parameter has on two model variables: the cytoplasmatic concentrations of TNF-α and IL-10. These results reveal that the two parameters governing IL-10 and TNF-α translation, referred to as *il10trans_new* and *tnfaalfatrans_new* in figure, have the greatest influence on the secretion of IL-10 and TNF-α in the cytoplasm, respectively. Instead, the parameter associated with intracellular cytokine degradation, *Dn*, impacts the production of both cytokines.

A qualitative sensitivity analysis was also conducted to test the robustness of the MFF AB model's state variables to variation of some key parameters. We found a strong

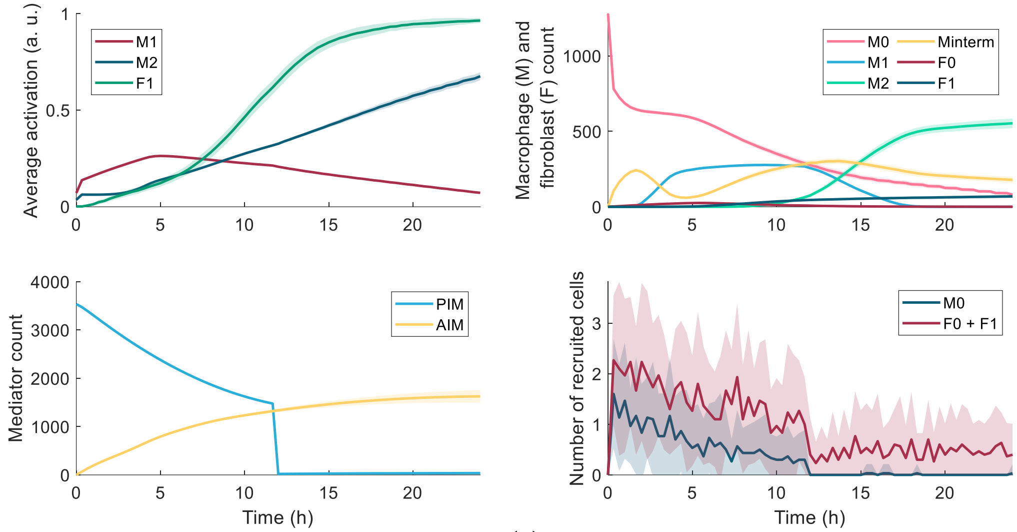

(a)

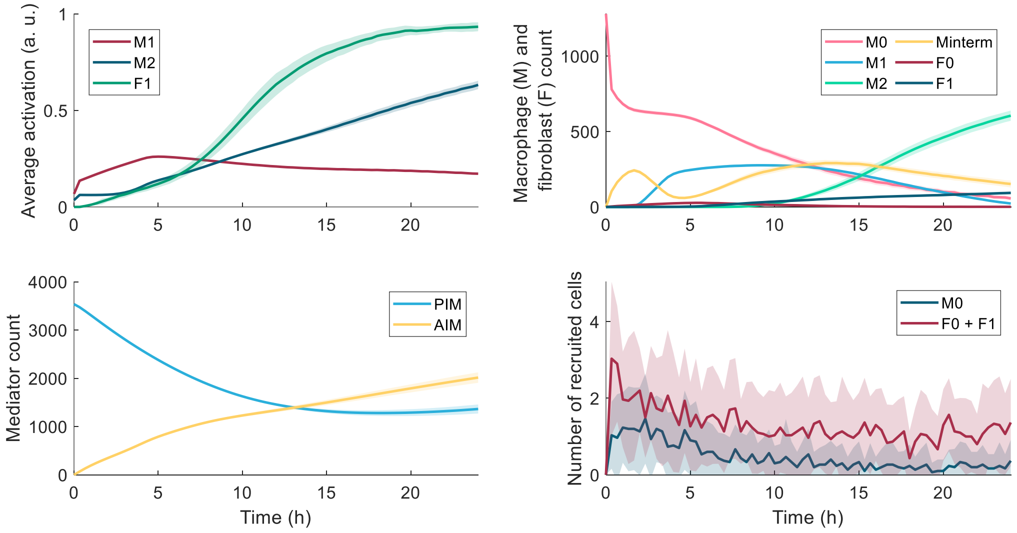

(b)

Fig S1. PIM washout scenario (a) versus continuous PIM exposure (b). Both in (a) and (b), from left to right and from top to bottom: activation variables, total counting of cells, amount of mediators and number of recruited cells.

dependence of the kinetics of secreted PIM and AIM on the parameter regulating effectiveness of AIM in inhibiting M1 activation of local cells by PIM (named M1AIMInfinity in ref. [1]), and on the rates at which fibrocytes and fibroblasts produce PIM and AIM (FibroProInflammatoryRate and FibroAntiInflammatoryRate, respectively). Fig S3 shows these kinetics, obtained by varying the above-mentioned parameters, running the

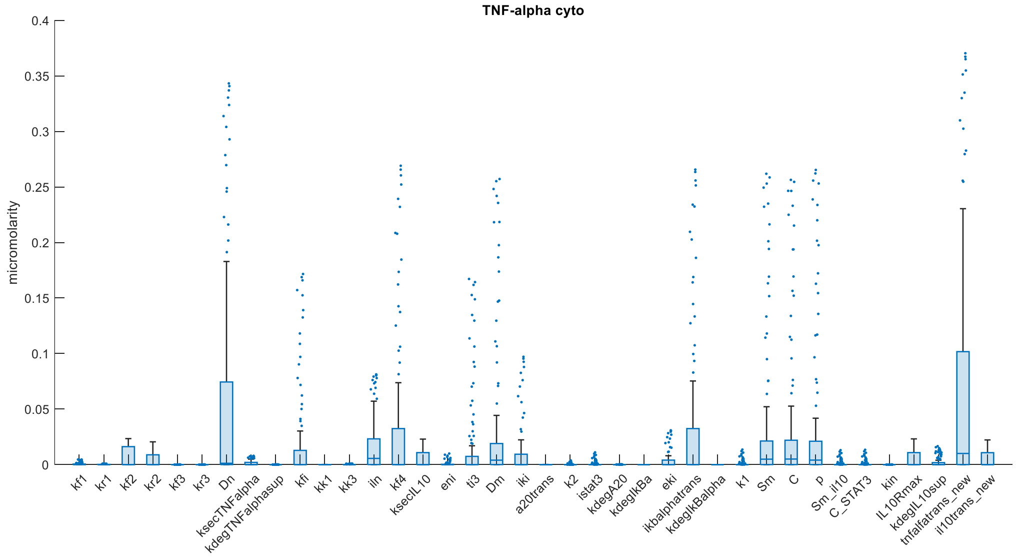

(a)

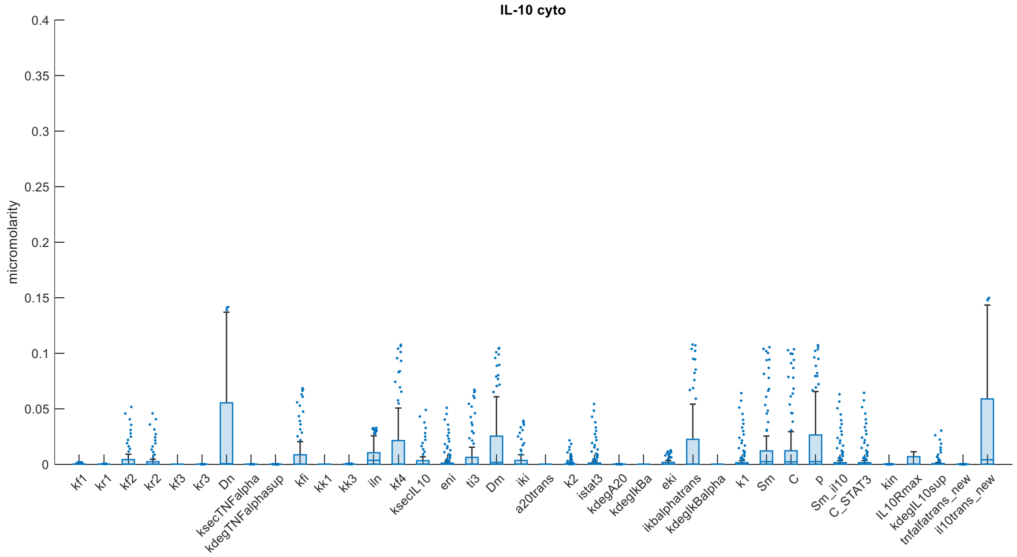

(b)

Fig S2. Bar plots illustrating the results of the sensitivity analysis, highlighting the relative impact of each parameter of the ODE model proposed by Maiti on the cytoplasmatic concentration of TNF-α (a) and IL-10 (b).

simulation for 144 hours, assuming M0 = 1280 resident macrophages and a chemical PIM as a stimulus with maximum value of 30.

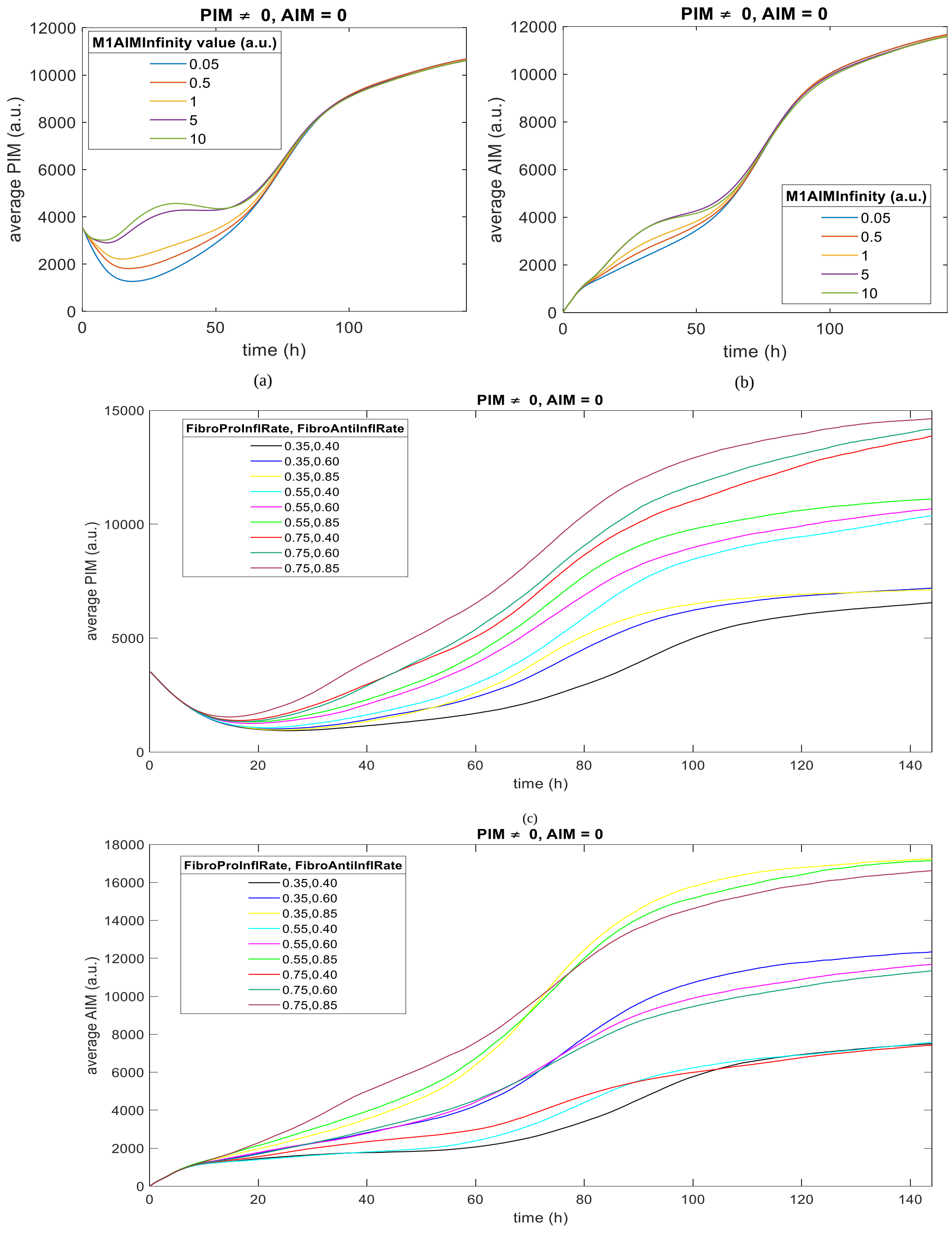

Fig S3. Average trends of PIM (panels a and c) and AIM (panels b and d) obtained by stimulating the system with a pro-inflammatory mediator and varying the following parameters: M1AIMInfinity (panels a and b), and the pair FibroProInflammatoryRate, FibroAntiInflammatoryRate (panels c and d).

References

1. Minucci SB, Heise RL, Reynolds AM. Agent-based vs. equation-based multi-scale modeling for macrophage polarization. PLoS ONE. 2024;19(1):e0270779.
