# Supplementary material for: Agent-based modeling of macrophage-fibroblast interactions in the immune response to biomaterials
Source: PLoS One. 2025 Aug 19;20(8):e0329186. doi: 10.1371/journal.pone.0329186 (PMC12364366; doi:10.1371/journal.pone.0329186)
Supplement: Attachment — Submitted filename: response_to_rev.pdf [file pone.0329186.s002.pdf]

Revision of the manuscript PONE-D-25-25184

Response to comments of Academic Editor

We thank the Academic Editor for the helpful comments, which we addressed as explained in the following paragraphs.

2. "Foreign body reaction (FBR)": full term for this abbreviation should be given at the first appearance in the main text. This applies to other abbreviations.

Thank you for this observation. In the revised version of the manuscript, we introduced the full term of each abbreviation used at its first appearance in the main text, including, among others, foreign body reaction (FBR), interleukin-12 (IL-12) and interferon-gamma (INF-γ).

2. Lines 541-543: "Macrophages, fibrocytes and fibroblasts are some of the key cells in the response to many inflammation conditions triggered by a pathogen, as well as in a FBR occurring when a biomaterial is implanted": there are no references to support this statement. More references should be cited, with this one (PMID: 38793665) as an example (citing is optional).

We thank the Academic Editor for this suggestion. In the new version of the manuscript, we added six references to support the reported statement in the Discussion session, including the one suggested in the comment, as follows:

Macrophages, fibrocytes and fibroblasts are some of the key cells in the response to many inflammation conditions triggered by a pathogen [14-16], as well as in a FBR occurring when a biomaterial is implanted [17 –19].

The new references are listed below:

14. M. Lech and H. J. Anders. Macrophages and fibrosis: How resident and infiltrating mononuclear phagocytes orchestrate all phases of tissue injury and repair. *Biochimica et Biophysica Acta (BBA) – Molecular Basis of Disease*, 1832(7):989–997, Jul 2013.

15. M. Weidenbusch and H. J. Anders. Tissue microenvironments define and get reinforced by macrophage phenotypes in homeostasis or during inflammation, repair and fibrosis. *J Innate Immun*, 4(5-6):463–477, Apr 2012.

16. B. M. Liu, N. Y. Rakhmanina, Z. Yang, and M. I. Bukrinsky. Mpox (monkeypox) virus and its co-infection with HIV, sexually transmitted infections, or bacterial superinfections: Double whammy or a new prime culprit? Viruses, 16(5):784, May 2024.

17. C. E. Witherel, D. Abebayehu, T. H. Barker, and K. L. Spiller. Macrophage and fibroblast interactions in biomaterial-mediated fibrosis. *Adv. Healthcare Mater.*, 8(4):1801451–1801451, Jan 2019.

18. R. Li, D. Feng, S. Han, X. Zhai, X. Yu, Y. Fu, and F. Jin. Macrophages and fibroblasts in foreign body reactions: How mechanical cues drive cell functions? *Materials Today Bio*, 22(100783):1–12, Oct 2023.

19. C. Chu, L. Liu, S. Rung, Y. Wang, Y. Ma, C. Hu, X. Zhao, Y. Man, and Y. Qu. Modulation of foreign body reaction and macrophage phenotypes concerning microenvironment. *J Biomed Mater Res.*, 108(1):127–135, Jan 2020.

Revision of the manuscript PONE-D-25-25184

Response to comments of Reviewer #1

In Maiti ode: "Three sets of data obtained for 0, 0.1 and 1 μg/mL LPS stimulations are used for parameter estimation, and the fourth dataset for 10 μg/mL LPS stimulation is used for model validation". Could the authors clarify the rationale for selecting 1 μg/mL LPS as the reference condition?

We thank the Reviewer for the question. We selected the value 1 μg/mL LPS as the reference condition since murine macrophages were stimulated with the same concentration in our experiments, as reported in subsection 2.1.1 of the manuscript:

"*The two ODE models were stimulated with the same LPS concentration as in the experiments (1 μg/mL).*".

Figure 3: The FACS data appear to include only six values. Are these values from technical or biological replicates? Clarification is needed.

Thank you for this comment. The values presented in Figure 3 correspond to the 6 different time points at which the FACS analysis was performed and are the average over 3 technical replicates of the same cell culture under identical experimental conditions. Specifically, for each sample, we performed three independent FACS measurements. The technical uncertainty is minimal (maximum variation ~1%), indicating high reproducibility of the instrument. In contrast, when performing biological triplicates, we observed a higher variability (~5disc–10%), as expected, since biological samples show higher intrinsic variability. We have included this information in the Figure 3 caption and in subsection 3.1 *Experimental validation of the pro-inflammatory dynamics of macrophages* of the *Results* section of the new version of the manuscript, respectively, as follows:

Fig 3. Correlation analysis between model (ODE and AB) and experiment (FACS) results. All the simulation variables and experimental measurements were reported in percentage of cells, coming from normalization and gating, respectively. To highlight that only six values from technical triplicates (maximum variation ~1%), in the FACS analysis were collected and used, respective curves have been dotted.

Fig 3 shows the percentage of the cytokine's production predicted by the ODE and AB models (where the former were computed with 1 μg/mL of LPS, as in the experimental setup), as well as the six values obtained from FACS experiments for the three different cytokines monitored (TNF-α, IL-12 and INF-γ). These represent technical replicates, obtained from repeated measurements of the same cell culture under identical experimental conditions. Specifically, for each sample, we performed three independent FACS measurements. The technical uncertainty is minimal (maximum variation ~1%), indicating high measurement precision. In contrast, when performing biological triplicates, we observed a higher intrinsic variability (~5–10%), as expected.

It would also be valuable to simulate conditions where LPS is washed out. As the current model only simulates conditions with continuous LPS exposure, it does not provide insights into the immune response following LPS removal. Simulations extending beyond 12 hours, especially post-stimulus, would be appreciated.

We appreciate the Reviewer's insightful suggestion. In response, we launched N = 30 runs with the MFF AB model for 24 hours, including a condition in which all the PIM in the lattice is removed, after stimulating the

system for 12 hours, thereby mimicking a complete washout scenario. This modification allows us to understand how the system adapts the inflammatory response after the stimulus is removed. For sake of comparison, we considered the kinetics of PIM and AIM, a well as the average activation variables, the counting of the cells involved and the number of newly recruited cells, for the two different cases, *without* and *with* washout. In both scenarios, we assumed 1280 resident non-activated macrophages and included the recruitment of new M0, F0 and F1 cells. Therefore, the only difference between the two simulations is in the different exposure to PIM. All the plots of the average variables are reported below:

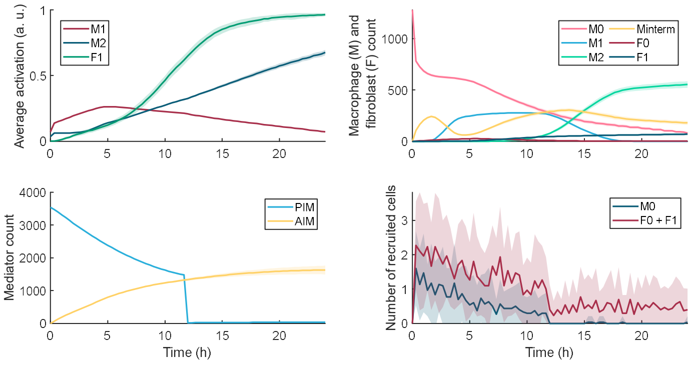

(a)

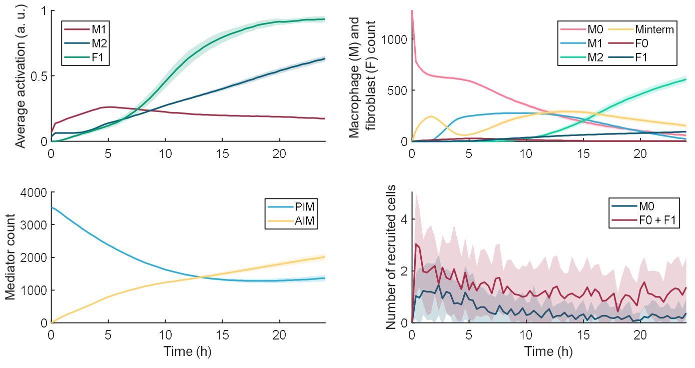

(b)

Figure S1. PIM washout scenario (a) versus continuous PIM exposure (b). Both in (a) and (b), from left to right and from top to bottom: activation variables, cell counting variables, mediators amount and number of recruited cells.

Interestingly, we observed comparable kinetics profiles between the continuous PIM stimulation and the washout condition, indicating that the system response remained largely unaffected by the removal of PIM at the selected time point. This indicates that a 12 hours PIM exposure is sufficient to trigger a self-sustaining inflammatory cascade, maintained through local agent interactions and intrinsic feedback mechanisms. This behaviour highlights the robustness of the model in capturing key aspects of the immune response, as the dynamics remain stable even in the absence of external stimulation. It is likely that a much shorter exposure of the system to the PIM stimulus would affect more extensively the cell dynamics. We have now added the above figures in the Supplementary Materials with the following text:

"Fig S1 shows, from left to right and from top to bottom, M1, M2 and F2 activation variables, the total number of cells, the amount of pro- and anti-inflammatory mediators and the number of newly recruited cells, in two different scenarios, a washout (a) and a continuous exposure of PIM (b). Notably, we found that the kinetics profiles were similar between continuous PIM stimulation and the washout condition, suggesting that the system's response was largely unchanged by the removal of PIM at the chosen time point. This suggests that a 12-hour exposure to PIM is sufficient to initiate a self-sustaining inflammatory cascade, driven by local interactions and internal feedback mechanisms. Such behaviour highlights the robustness of the model in capturing essential features of the immune response, as the dynamics remain stable even without ongoing

external stimulation. However, it is likely that a significantly shorter exposure to PIM would more substantially alter cellular dynamics."

Biological Variability, when validating, are cells all murine cells? cell heterogeneity would benefits the model by modeling an "average" cell or system.

We agree with the Reviewer that including a broader range of cell types beyond murine cells would enhance the biological variability and generalizability of the model. Unfortunately, we only had access to murine cell data. This homogeneity may restrict the extent to which the model reflects interspecies or intercellular diversity. However, it is worth noting that some of the intrinsic parameter values used in our model, taken from Minucci's AB model, were tuned so that it returns similar kinetics to those observed with Maiti's ODE model (see ref. [3] of the new version of the manuscript for more detail). This model includes, among others, parameters experimentally derived using murine cells from different organs, thus implicitly incorporating a degree of biological variability. Such information has been derived from the references included in Table 2 of ref. [8] in the new version of the manuscript. As an example, below we report one of them, where rat liver hepatoma cells were used:

- Z. Huang, F. Senocak, A. Jayaraman, and J. Hahn. Integrated modeling and experimental approach for determining transcription factor profiles from fluorescent reporter data. *BMC Syst Biol* 2(64), Jul 2008.

Therefore, while our own dataset lacks cellular heterogeneity, the model may still capture certain aspects of biological variability through the parameters employed.

Additionally, how does the model distinguish biological noise (natural variability in cytokine production or cellular response) from technical noise (e.g., experimental error, measurement artifacts)? A discussion on how this was handled—e.g., through replicates, probabilistic rules in the agent-based model, or sensitivity analysis—would strengthen the validation and interpretation of model robustness.

We thank the Reviewer for this important point regarding the distinction between biological variability and inconsistencies introduced by the experimental or measurement process. Given their distinct nature, this issue has been differently addressed in the ODE-based and AB modelling frameworks. The ODE models are deterministic by design. Therefore, to account for biological variability, we performed a simple sensitivity analysis at the beginning of this study, to explore how variations in key parameters affect the cytoplasmatic production of pro- (TNF-α) and anti- (IL-10) inflammatory cytokines in Maiti's model, running it for 12 hours. Our results suggested that the two parameters describing IL-10 and TNF-α translation affect the production of cytoplasmatic IL-10 and TNF-α the most, respectively. The parameter indicating the degradation of intracellular cytokine affect instead the production of both cytokines. Below you can find the result of our sensitivity analysis displayed in a bar plot, where cytokine concentrations have been expressed in micromolarity (μmol).

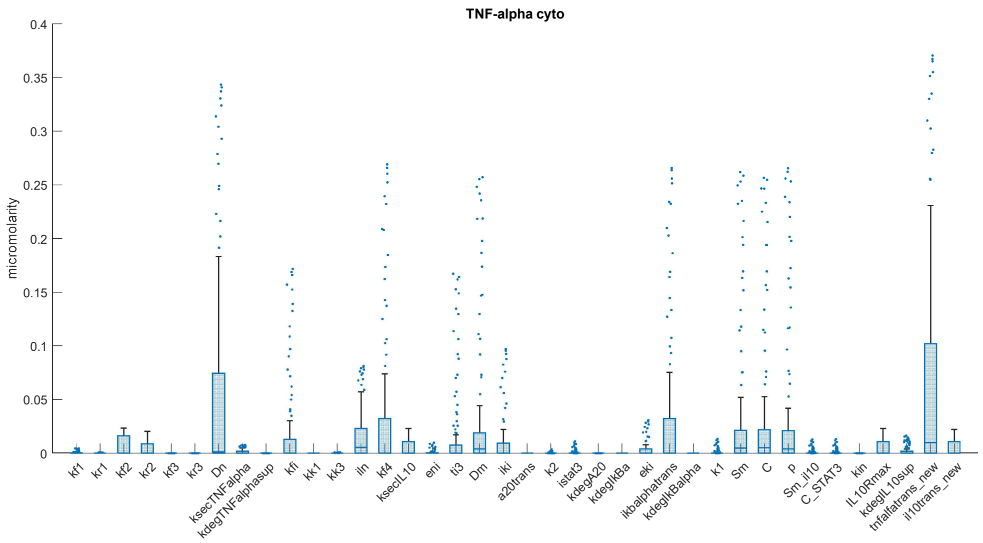

(a)

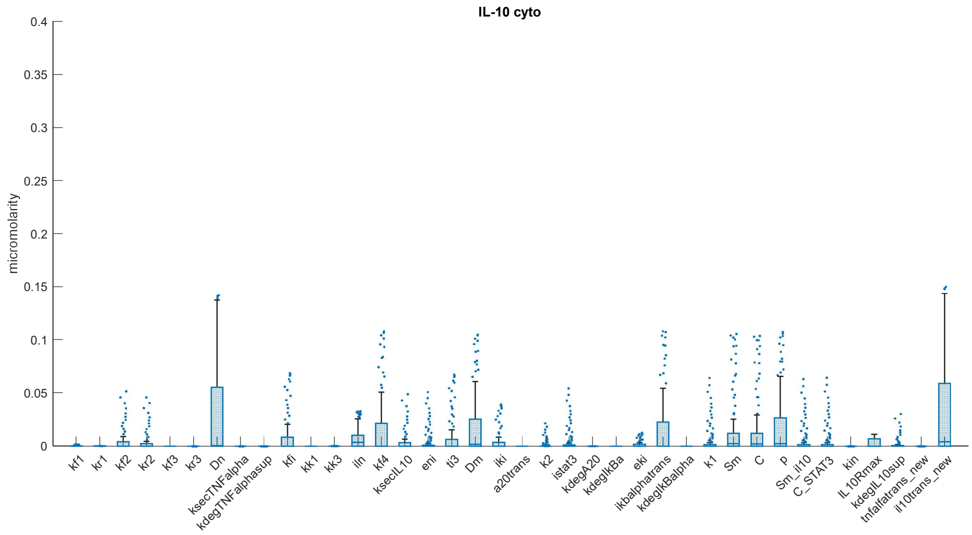

(b)

Fig S2. Bar plots illustrating the results of the sensitivity analysis, highlighting the relative impact of each parameter of the ODE model proposed by Maiti on the cytoplasmatic concentration of TNF-α (a) and IL-10 (b).

In the Supplementary Materials included in the new version of the manuscript, you can find this figure, and the following text to discuss it:

"Bar plots in Fig S2 represent the outcomes of the sensitivity analysis performed for the ODE model by Maiti, with each bar indicating the magnitude of influence that individual parameters have on the model's behaviour. More in detail, we evaluated the effect that the variation of each parameter has on two model variables: the cytoplasmatic concentrations of TNF-α and IL-10. These results reveal that the two parameters governing IL-10 and TNF-α translation, referred to as il10trans_new and tnfaalfatrans_new in figure, have the greatest influence on the secretion of IL-10 and TNF-α in the cytoplasm, respectively. Instead, the parameter associated with intracellular cytokine degradation, Dn, impacts the production of both cytokines."

Regarding technical noise, it was not explicitly included in this particular ODE framework, but any kind of technical error can be theoretically modelled, thus simulating measurement errors or experimental conditions.

In contrast, in the AB models (both the Minucci's version and the MFF proposed in the manuscript), biological noise is explicitly incorporated through probabilistic rules, which reflect natural variability in cytokine production and cell responses in each agent. This effect of the biological randomness was analyzed by conducting multiple replicates to identify consistent patterns and distinguish them from random fluctuations. In addition, we also conducted a sensitivity analysis to test how robust the MFF AB model's state variables are to variation in parameters. We found a strong dependence of the PIM and AIM kinetics on the parameter regulating effectiveness of AIM in inhibiting M1 activation of local cells by PIM (*M1AIMInfinity* in ref. [3]), and on the rates at which fibrocytes and fibroblasts produce PIM and AIM (*FibroProInflammatoryRate* and *FibroAntiInflammatoryRate*, respectively). Below, you can find the trends of PIM and AIM obtained by varying the above-mentioned parameters, running the simulation for 144 hours, assuming M0 = 1280 resident macrophages and a chemical PIM as a stimulus with maximum value of 30. These figures have been included in the Supplementary Materials of the manuscript, with the following paragraph to elucidate them:

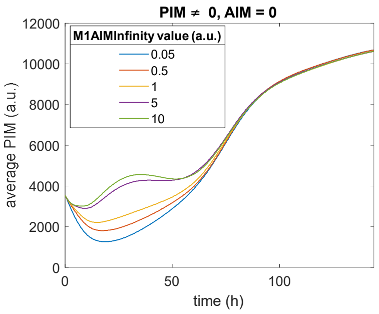

(a)

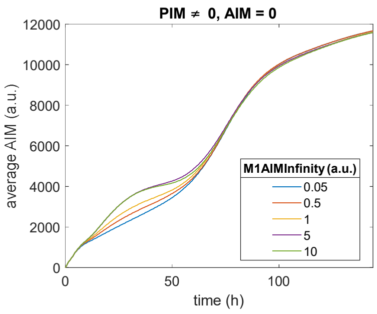

(b)

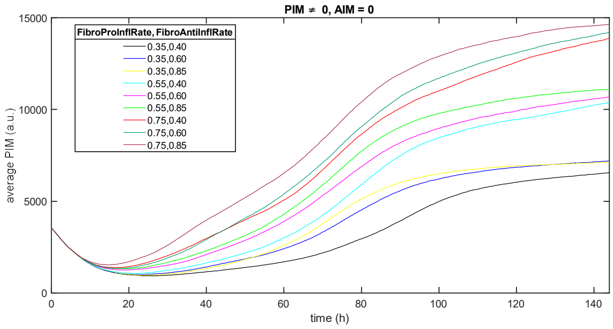

(c)

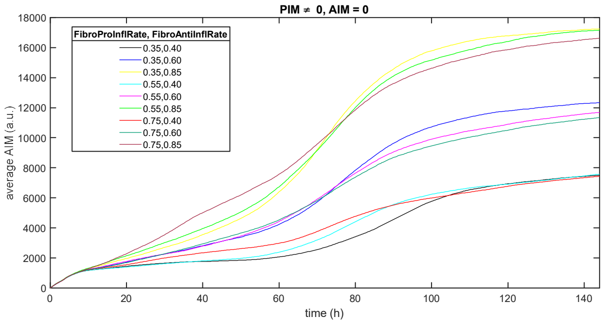

(d)

Fig S3. Average trends of PIM (panels a and c) and AIM (panels b and d) obtained by stimulating the system with a pro-inflammatory mediator and varying the following parameters: M1AIMInfinity (panels a and b), and the pair FibroProInflammatoryRate, FibroAntiInflammatoryRate (panels c and d).

"A qualitative sensitivity analysis was also conducted to test the robustness of the MFF AB model's state variables to variation of some key parameters. We found a strong dependence of the kinetics of secreted PIM and AIM on the parameter regulating effectiveness of AIM in inhibiting M1 activation of local cells by PIM (named M1AIMInfinity in ref. [1]), and on the rates at which fibrocytes and fibroblasts produce PIM and AIM (FibroProInflammatoryRate and FibroAntiInflammatoryRate, respectively). Fig S3 shows these kinetics, obtained by varying the above-mentioned parameters, running the simulation for 144 hours, assuming M0 = 1280 resident macrophages and a chemical PIM as a stimulus with maximum value of 30."

As in the former case, technical noise is not contemplated in the AB models. These are designed to simulate the behaviours of agents within a system, not the inaccuracies of the data collection process. Technical noise depends on the measurement tool chosen, which is also dependent on which variable one decides to measure. Given that multiple options might exist, technical noise is intrinsically hard to model, and not univocally defined.

The current validation relies heavily on results from a limited source. To improve robustness, the model should be tested against a broader range of experimental datasets. This will help ensure the model is not overfitted to a specific scenario and performs reliably across diverse conditions.

We are aware of the limited source of experimental data used for the validation of the proposed MFF model. We found a large amount of papers containing potentially useful experimental datasets, some of which are listed below:

1) Barcena M. L., Niehues M. H., Christiansen C., Estepa M., Haritonow N., Sadighi A. H., *et al.*, Male Macrophages and Fibroblasts from C57/BL6J Mice Are More Susceptible to Inflammatory Stimuli. *Front. Immunol*. Nov 2021, 12:758767.
2) Sundararaj K. P., Samuvel D. J., Li Y., Sanders J. J., Lopes-Virella M. F., and Huang Y., Interleukin-6 released from fibroblasts is essential for up-regulation of matrix metalloproteinase-1 expression by U937 macrophages in coculture: cross-talking between fibroblasts and U937 macrophages exposed to high glucose. *J Biol Chem*. May 2009, 15;284(20):13714-13724.
3) Le Fournis C., Jeanneau C., Giraud T., El Karim I., Lundy F., and About I. Fibroblasts Control Macrophage Differentiation during Pulp Inflammation. *Journal of Endodontics*, Sep 2021, 47(9):1427-1434.

However, they did not allow a direct and quantitative comparison with our types of results, which are expressed in terms of state variables representing cytokine and cell quantification, as well as the activation of cells. As an example, in [2], the experimental data are extracted using ELISA and Real Time PCR, aiming at quantifying supernatant and mRNA cytokines, respectively, in human cells.

Overall, this model is a good step but need more improvement on feeding more experimental data and avoid overfitting to one situation. The future of this model is bright but current immune profile of FBR can be done by wet lab alone.

We thank the Reviewer for this concluding comment. We are aware that the AB MFF model is in its early stage, and that the long-term aim of developing a digital twin of the FBR can only be achieved by validation with more biological data.

References
